# Evolution of regulatory system in India

**DOI:** 10.4103/0253-7613.58507

**Published:** 2009-10

**Authors:** Shiv Prakash

**Affiliations:** Chief Editor, Indian Journal of Pharmacology, editor@IJP-online.com

This decade has given the Pharmaceutical industry a new perspective. Notable of these is the phenomenal growth of Contract Research Organization (CRO) industry and the emergence of India as an important clinical research destination in the world. This has made the international sponsors to expect more from regulatory body. Also, the growth of pharmaceutical companies has resulted in exceptional increase in exports to regulated markets across the globe. However the regulatory system has not kept the pace with the growth of the industry.

The expectation form the regulatory system is innovation on its part. When David Kessler took over as FDA Commissioner in 1990, the average time for any drug approval was 3 years. He changed the outlook of the FDA by putting life-saving drugs on fast track approval. For instance, it took just 45 days in 1996 for the approval of protease inhibitors. This made the life-saving drugs easily available to HIV-affected people and brought about a revolution in combating these deadly diseases. The regulatory system became transparent informing people about benefits as well as risks and ended up being right. Under the right leadership, even the conservative government organizations such as the Drugs Controller General of India (DCGI) can take informed risks and make a huge difference.

There are many steps that DCGI has taken in recent past that has set good examples in a right direction for the industry. Registration of CROs with DCGI allows them to know who is doing what and can authenticate CROs in India. Decision to check the sites while trials also helps DCGI check the compliance with the trial. Registration of clinical trials has made the conduct of clinical trials more transparent. Also access to the new drugs is made easy for those people who would want to participate and undergo treatment with new drugs. The evolving regulatory system may not be apprehensive or hesitant in future to allow Phase I studies to be conducted in India for all the drugs.

There are some realities to face for the rapid development of regulatory system to match up with the pace at which CRO industry is growing. The DCGI is understaffed and lacks the expertise to evaluate protocols. As a result, persistent follow-up, including personal visits to the DCGI, is required in order to “push” an application for a trial forward. On the other hand, although the country has more than half a million practicing doctors, fewer than 200 investigators have been trained in good clinical practice. Among around 14,000 general hospitals, no more than 150 have the adequate infrastructure to conduct trials, and there are fewer than a dozen pathology laboratories that meet the criteria for compliance with good laboratory practice and good clinical practice. Smaller and ill-equipped pathology labs also declare themselves as central labs which lead to generation of unreliable lab results for the study. Only about half of the larger hospitals have Institutional Review Boards. Many of these boards have not yet formulated standard operating procedures and they often lack the expertise to evaluate protocols. Information about conflicts of interest is neither sought nor voluntarily provided by investigators.

Many aspects in India are not regulatory intensive, these may lead to supply of poor quality pharmaceutical agents to the patients. There is no way to check the quality of drugs after 4 years from the date of first introduction in India. During this 4-year period the drugs will be under “new drug” category and they require bioequivalence and if necessary clinical studies are conducted. Those drugs which are called “old drugs” after this 4-year period, need not be subjected to bioequivalence studies and they can be permitted to market without these stringent requirements as compared to new drugs. The permission can be easily obtained from local state authorities. It is time to consider to change the “new drug” definition in Drugs and Cosmetics Act. Biological drugs or biosimilars are registered easily (for example many brands of Insulin and EPO), whereas it takes years and number of clinical studies to register these in Europe and USA. Permission to sell irrational combinations without any credible data is another menace.

Evolution of regulatory system changes the industry in any country and encourages people to do more to discover and invent new drugs for emerging diseases. India is rich in biodiversity and plants with high medicinal values. We are a failure in capitalizing in encouraging industry and research institutes to create medicines from natural resources. A regulatory system to tap this potential would have helped our country to do great in the nutraceutical business. This is an example how the non-existent or poor regulatory system tapping the natural wealth of any country can make it non-competitive in the world market. An encouraging regulatory approach to create more evidence to our natural wealth would create new markets globally and can stimulate innovation in evidence-based medicine based on Indian Plants.

The USFDA is responsible for giving rise to the most competitive pharmaceutical industry in the world. They set standards so that doctors and patients are not afraid of using new drugs.

A similar speedy, vigilant, and evolving robust regulatory system is in the best interest of India.

